# Theory and strategy for Pneumococcal vaccines in the elderly

**DOI:** 10.1080/21645515.2015.1075678

**Published:** 2015-09-25

**Authors:** Ho Namkoong, Makoto Ishii, Yohei Funatsu, Yoshifumi Kimizuka, Kazuma Yagi, Takahiro Asami, Takanori Asakura, Shoji Suzuki, Testuro Kamo, Hiroshi Fujiwara, Sadatomo Tasaka, Tomoko Betsuyaku, Naoki Hasegawa

**Affiliations:** 1 Division of Pulmonary Medicine Department of Medicine; Keio University School of Medicine; Tokyo, Japan; 2 Center for Infectious Diseases and Infection Control; Keio University School of Medicine; Tokyo, Japan

**Keywords:** aging, elderly, pneumococcal vaccines, pneumococcal conjugate vaccine, pneumococcal polysaccharide vaccine, pneumococcal diseases, senescence

## Abstract

Pneumonia is the fourth-leading cause of death globally, and *Streptococcus pneumoniae* is the most important causative pathogen. Because the incidence of pneumococcal diseases is likely to increase with the aging society, we should determine an optimal strategy for pneumococcal vaccination. While consensus indicates that 23-valent pneumococcal polysaccharide vaccine prevents invasive pneumococcal diseases (IPD), its effects on community-acquired pneumonia (CAP) remain controversial. Recently, a 13-valent pneumococcal conjugate vaccine (PCV13) was released. The latest clinical study (CAPiTA study) showed that PCV13 reduced vaccine-type CAP and IPD. Based on these results, the Advisory Committee on Immunization Practices recommended initial vaccination with PCV13 for the elderly. Scientific evidence regarding immunosenescence is needed to determine a more ideal vaccination strategy for the elderly with impaired innate and adaptive immunity. Continuing research on the cost effectiveness of new vaccine strategies considering constantly changing epidemiology is also warranted.

## Abbreviations

CAP: community-acquired pneumonia
HAP: hospital-acquired pneumonia
VAP: ventilator-associated pneumonia
IPD: invasive pneumococcal diseases
PPV23: 23-valent pneumococcal polysaccharide vaccine
PCV13: 13-valent pneumococcal conjugate vaccine
CAPiTA: Community-Acquired Pneumonia Immunization Trial in Adults
ACIP: Advisory Committee on Immunization Practices
OI: opsonisation index
AID: activation-induced cytidine deaminase
PCV7: 7-valent pneumococcal conjugate vaccine
IL: interleukin.

## Introduction

Pneumonia is the fourth-leading cause of death globally, according to the World Health Reports 2014 by the World Health Organization.^1^ In developed countries, such as Japan, more than 95% of deaths from pneumonia occur in the elderly (≥65 years old). Therefore, faced with the increasing aging population in both developed and developing countries, it is becoming more important to prevent pneumonia.^2^

In terms of clinical settings, pneumonia can be classified into several categories such as community-acquired pneumonia (CAP), hospital-acquired pneumonia (HAP), ventilator-associated pneumonia (VAP), or healthcare-associated pneumonia.^3,4^ In addition, based on etiology, pneumonia could be categorized as aspiration pneumonia or post-influenza pneumonia.^5,6^ *Streptococcus pneumoniae* (*S. pneumoniae*) is the leading bacterial cause of CAP, aspiration pneumonia, and post-influenza pneumonia ^7,8^ and also one of the major causative bacteria of HAP and VAP.^9^ Therefore, to reduce the disease burden of pneumonia in the aging society, infections with *S. pneumonia* should be addressed; the introduction of pneumococcal vaccination decreases the relative occurrence of pneumococcal infections, compared with other causative bacteria.^10,11^ In this review, we aimed to review new evidence for pneumococcal vaccines from both basic and clinical points of view to build an optimal vaccine strategy.

## Serotype of Streptococcus Pneumoniae

The morphological characteristics of *S. pneumoniae* include a polysaccharide capsule that surrounds a diplococci of *S. pneumoniae*.^12^ There are more than 90 serotypes of *S. pneumonia* based on the type of capsule, on which the antigenicity also chiefly depends.^13^ Among the various pathogenic factors including neuraminidase and pneumolysin, the capsule is the most important barrier by which binding of the host's complement to the surface of the bacteria is inhibited, allowing escape from opsonisation.^14,15^ Based on this feature, different *S. pneumoniae* serotypes have different pathogenicities, including colony forming ability, severity, invasiveness, and drug resistance profile.^16,17^ For instance, serotype 3 is one of the most invasive serotypes since it has the thickest capsule of all *S. pneumoniae* serotypes.^18,19^

### Invasive pneumococcal diseases, non-invasive pneumococcal diseases, and pneumococcal pneumonia

**Figure 1.**
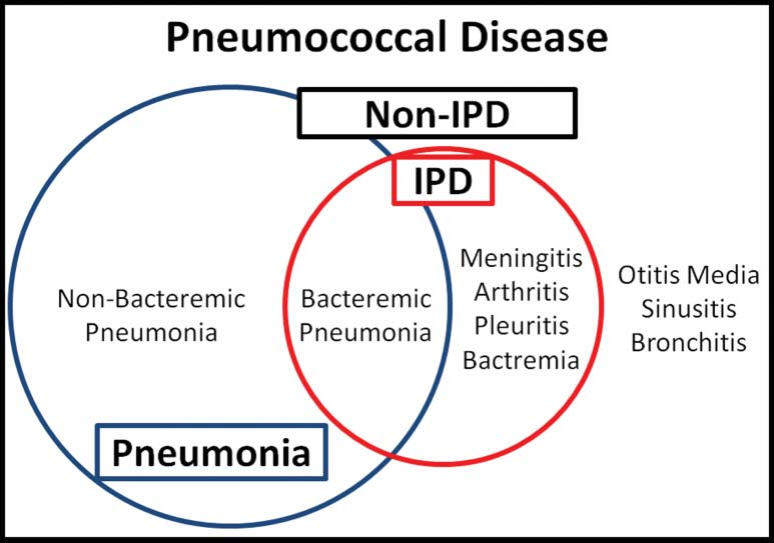
**Schematic spectrum of pneumococcal disease** Pneumococcal diseases consist of 2 groups: invasive pneumococcal disease (IPD) and non-IPD. IPD is a pneumococcal disease in which *Streptococcus pneumoniae* is detected in a sterile space. IPD consists of bacteraemic pneumonia, pleuritis, meningitis, arthritis, and bacteraemia, and non-IPD consists of non-bacteraemic pneumonia, otitis media, sinusitis, and bronchitis. Although bacteraemia occurs in only 10–30% of pneumococcal pneumonia cases, bacteraemic pneumonia is the most common IPD owing to the clinical frequency of pneumococcal pneumonia. In clinical settings, pleuritis usually occurrs with pneumonia.

In invasive pneumococcal diseases (IPDs), *S. pneumoniae* is detected in sterile sites such as cerebrospinal fluid, pleural fluid, joint fluid, and the blood (**Fig. 1**).^20^ Although bacteraemia occurs in only 10–30% of pneumococcal pneumonias, bacteraemic pneumonia is the most common IPD owing to the clinical frequency. On the other hand, non-IPD usually includes non-bacteraemic pneumococcal pneumonia, bronchitis, sinusitis, and otitis media.

It is important to understand the differences between IPDs and non-IPDs. First, the distribution of *S. pneumoniae* serotypes is epidemiologically different as with the case of pathogenicities.^21-23^ Moreover, the geographical serotype distribution could change chronologically with the vaccine introduction and its gradual spread, even in the same region.^16^ Therefore, assessing the epidemiological distribution of both IPD and non-IPD is the important initial action at the time of introducing pneumococcal vaccination. Second, the disease severity is different; despite a considerably lower incidence of IPD than pneumococcal pneumonia, the prognosis is poorer, and the aftereffects result in greater patient burden.^24^ In this context, both pneumococcal pneumonia and IPD are clinically meaningful.

### Characteristics of the 23-valent pneumococcal polysaccharide vaccine (PPV23) and 13-valent pneumococcal conjugate vaccine (PCV13)

The 23-valent pneumococcal polysaccharide vaccine (PPV23) is an inactivated vaccine that is refined from polysaccharide capsules of 23 types of *S. pneumoniae*.^25^ The polysaccharide antigen of capsules induces only B-cell immunity, not T-cell immunity, resulting in a short lasting immune response and lack of memory immunity (**Table 1**).^26^ The activity of the serotype-specific antibody and opsonisation decreases approximately 5 y after vaccination; therefore, re-vaccination is recommended in many countries and regions.^27^ However, booster effects might be limited because of hyporesponsiveness associated with a depletion of the memory B-cell population.^28^

**Table 1.** Comparison between 23-valent pneumococcal polysaccharide vaccine (PPV23) and 13-valent pneumococcal conjugate vaccine (PCV13)

	PPV23	PCV13
Characteristics	Inactive vaccine made of a capsular polysaccharide	Inactive vaccine made of a capsular polysaccharide combined with diphtheria toxoid
Strengths	Wider coverage of serotypes Long history of experience	T-cell dependent immunity Memory immunity is evoked Booster effect and herd immunity are expected
Limitations	T-cell independent immunity Lack of memory immunity	Narrower coverage of serotypes Less evidence of cost effectiveness

However, this phenomenon is controversial because antigen-specific opsonisation is sufficiently evoked even by revaccination with PPV23, which is more closely related to immunogenicity rather than antigen-specific IgG levels.^29^ Regarding more data on hyporesponsiveness of PPV23, Hammitt et al. studied the immunogenicity of PPV23 among adults aged 55–74 y who were administered up to 4 doses of PPV23. The IgG titer levels and opsonisation index (OI) were measured 30 d after vaccination.^30^ Hyporesponsiveness was not observed with repeated PPV23 vaccination. Also, Musher et al. also reported that IgG concentrations still exceeded vaccine-naïve levels 10 y after revaccination and that the second and third doses were potently immunogenic.^31^ Available data for immunogenicity following the 13-valent pneumococcal conjugate vaccine (PCV13) vaccination in adults are limited at the present because of a brief history since the introduction. Therefore, we should keep updating with data for a long-term immunogenicity for those who receive the PPV23 revaccination and forthcoming head-to-head data comparing immunogenicity between PPV23 and PCV13.

PCV13 is also an inactivated vaccine as PPV23, but it is capable of activating both B-cell and T-cell immune responses through the action of diphtheria toxoid binding to the polysaccharide leading to the induction of a sufficient immune reaction even in infants who have immature B-cell immunity.^32,33^ For this reason, PCV13 is licensed in many countries for infants.^34^ In addition, efficacy has been demonstrated in high-risk adults because of the evoked T-cell immune response and enhanced memory response with a booster vaccination.^35-37^ Thus, it has been reported that in individuals with HIV, chronic obstructive pulmonary disease, or renal transplantation, PCV13 evokes better immunogenicity than PPV23 in a subset of serotypes.^38-41^

PCV13 also reportedly reduces nasopharyngeal carriage of the vaccine type of pneumococci, especially in children.^42^ The incidence of pneumococcal disease in adults is reportedly reduced in some regions owing to herd immunity with PCV13, since nasopharyngeal carriage of pneumococci in adults can be transmitted from children.^43,44^

Regarding serotype coverage, PPV23 contains polysaccharides of pneumococcal serotypes 1, 2, 3, 4, 5, 6B, 7F, 8, 9N, 9V, 10, 11A, 12F, 14, 15B, 17F, 18C, 19F, 19A, 20, 22F, and 23F, while PCV13 contains polysaccharides of pneumococcal serotypes 1, 3, 4, 5, 6A, 7F, 6B, 9V, 14, 18C, 19A, 19F, and 23F. Therefore, PPV23 has wider coverage than PCV13, although serotype 6A is covered only by PCV13. The most recent domestic reports from Japan of the serotype distribution of CAP and IPD indicate that the proportions of CAP serotypes covered by PPV23 and PCV13 are 62.7% and 49.3%, respectively, and the proportions of IPD serotypes covered by PPV23 and PCV13 are 69.6% and 48.0%, respectively. The pneumococcal serotype distribution have shifted from vaccine-type to non-vaccine-type serotypes since the introduction of PCVs.^45,46^ The more PPV23 and PCV13 become prevalent both in infants and adults, the further the coverage of vaccine-type serotypes is expected to decrease in the future. Therefore, we should monitor epidemiological information on serotype transition.

### Cochrane review of PPV23 and other clinical study on PPV23

The effectiveness of PPV23 for prevention of IPD in adults is widely accepted.^47^ The Cochrane review published in 2013 consistently showed strong evidence that PPV23 is effective for preventing IPD.^48^ A meta-analysis including 11 randomized controlled trials found that PPV23 was effective against IPD (odds ratio [OR] 0.26, 95% confidence interval [CI] 0.14–0.45, I^2^ statistic = 0%) with no statistical heterogeneity. Generally, I^2^ statistic values of 25%, 50%, and 75% indicate low, moderate, and high heterogeneity, respectively.

Regarding the effect of PPV23 on CAP, the Cochrane review does not provide compelling evidence to support the routine use of PPV23 for the prevention of all-cause pneumonia or mortality. Although efficacy against all-cause pneumonia was shown in low-income countries (OR 0.54, 95% CI 0.43–0.67, I^2^ statistic = 19%), it was not demonstrated in the general population (OR 0.71, 95% CI 0.45–1.12, I^2^ statistic = 93%) or adults with chronic illness (OR 0.93, 95% CI 0.73–1.19, I^2^ statistic = 10%) in high-income countries. Furthermore, PPV23 was not associated with substantial reductions in all-cause mortality (OR 0.90, 95% CI 0.74–1.09; random-effects model, I^2^ statistic = 69%). As indicated by the high I^2^ statistic values, there was obvious heterogeneity in those randomized clinical trials.

Although not included in the Cochrane review, the efficacy of PPV23 was supported in the CAPAMIS study, which was a population-based prospective cohort study involving 27,204 individuals aged >60 years in Spain.^49^ Among a total of 76,033 person-years, 29,065 (38%) person-years were assigned to immunized subjects. Although PPV23 did not appear to be effective in primary analyses, PPV23 vaccination administered within 5 y was associated with reduced risks for bacteraemic pneumococcal CAP, non-bacteraemic pneumococcal CAP, overall CAP, and all-cause CAP by analyses adjusted for multiple variables. Noteworthy, they proved a protective effect of recent PPV23 vaccination by focusing on the subgroup vaccinated within previous 5 y. This implies a protective effect of PPV23 last for about 5 y from the epidemiological aspect.

### CAPiTA study

In 2011, PCV13 was licensed for older adults under the United States Food and Drug Administration's accelerated approval program.^50^ The Netherlands-based Community-Acquired Pneumonia Immunization Trial in Adults (CAPiTA) was designed to meet regulatory commitments under the accelerated approval program and verify the clinical benefit of PCV13 ^51^ in the first episode of vaccine-type CAP in adults ≥65 years of age. This clinical trial excluded users of immunosuppressants and participants with potential nursing and healthcare-associated pneumonia. Reductions in vaccine-type IPD (75.00%; P = 0.0005), vaccine-type pneumococcal CAP (45.56%; P = 0.0006), and non-bacteraemic/non-invasive vaccine-type pneumococcal CAP (45.00%; P = 0.0067) were observed.^52^ However, the CAPiTA study did not demonstrate clinical efficacy of PCV13 for non-vaccine-type pneumococcal CAP. The safety profile of PCV13 in the CAPiTA study was consistent with studies previously conducted in adults.^53-55^

### Advisory Committee on Immunization Practices (ACIP) recommendations

The CAPiTA study demonstrated clinical efficacy of PCV13 for vaccine-type pneumococcal CAP, which was not supported as compelling evidence by the Cochrane review. From this study, the Advisory Committee on Immunization Practices (ACIP) announced recommendation in the Morbidity and Mortality Weekly Report issued in September 2014 that all adults ≥65 years of age should receive initially PCV13 followed subsequently PPSV23 in 6–12 months.^56^ This 2-step vaccination approach aims to maximize both the efficacy of pneumococcal vaccination in terms of immunogenicity developing acquired memory T-cell function by initial PCV13 and wider serotype coverage by subsequent PPV23. As a theoretical background for initial vaccination with PCV13, some clinical trials have demonstrated booster effects with PPV23 inoculation after PCV13 but not with PCV13 inoculation after PPV23. For example, elderly people receiving PPV23 one year after an initial 7-valent pneumococcal conjugate vaccine (PCV7) vaccination had 3-fold lower IgG and OI values than those who received PCV7/PCV7 or PCV7/PPV23 vaccinations.^57^

Notably, review of the ACIP recommendation is planned for 2018 owing to potential changes in the epidemiological situation.^56^ In particular, recent epidemiological studies have reported that IPD due to non-PCV13 serotypes has increased not only among infants but also among the elderly despite a dramatic reduction in IPD due to PCV-13 serotypes.^58-61^ Not only in IPD, the relative increase in non-bacteraemic pneumococcal pneumonia has been also observed among adults in some regions as ‘serotype replacement’.^62^ The distribution of pneumococcal diseases due to non-PCV13 is expected to increase more resulting from its direct effect and herd immunity effect; therefore, we should objectively re-evaluate the efficacy and cost-effectiveness based on the timely epidemiological data.

### Immunosenescence of pneumococcal vaccinations

Because the importance of pneumonia prevention is increasing with the aging population and the higher immunologic risk in elderly people, an optimal strategy for pneumococcal vaccination considering maximal immunogenicity is needed, for which we need to have a good understanding of the effects of aging on the fundamental immunological functions.^63^ Recent translational studies of immunological senescence, which is also called ‘immunosenescence’, have focused on both adaptive and innate immunity.^64-66^

Aging negatively affects T-cell immunity partly resulting from atrophy of both the thymic cortex and medulla, where T-cells are generated.^67^ In particular, the age-dependent reduction in the number of mature naïve T-cells causes elderly people to be more vulnerable to never encountered pathogens.^68^ In aged mouse models, antigen-inexperienced T-cells are functionally hampered and difficult to prime.^69^ Besides naïve T-cells, senescence results in decreased affinity of antigen-specific memory T-cells to antigens at the peripheral level, such as lymph nodes and the inflammation site.^70,71^ Although memory T-cells survive longer, proliferation and cytokine production are impaired during the recall phase.^69^

Senescence also dampens the function of B-cells primarily because of reduced interleukin (IL)-7 production, decreased activation-induced cytidine deaminase (AID) activity, and hampered transcriptional factor E47 activity.^72-75^ IL-7 is a cytokine that promotes the differentiation from pre-B-cells to B-cells and their migration from the bone marrow to the blood stream.^76^ Aging diminishes IL-7 production by depressing the function of bone marrow stroma cells.^77^ AID is an essential enzyme for the class switch of antibodies and causes higher affinity against antigens by modulating somatic hypermutation.^78^ Therefore, it is noted that impaired AID function owing to senescence results in immaturation of humoral immunity. In terms of innate immunity in the elderly, the reduction in the phagocytic capacity of neutrophils and macrophages,^79^ the impairment of up-regulation of major histocompatibility complex class II, and defects in the expression of toll-like receptors are known.^80,81^ Chronic inflammation in aging also decreases the ability to recognize danger signals provoked by vaccination.^82^ Furthermore, capacity of complements and natural killer-cells, which play a pivotal role in the protection against pneumococcal infection, have been also reported to be reduced with senescence.^81,83-86^

Taken together, aging results in insufficient immunogenicity in response to vaccination. In turn, it leads to increased susceptibility to pneumococcal infections. Moreover, age-dependent impaired function of IgGs, complements, and neutrophils ^87^ would fail to coordinate sufficient opsonisation which is one of the most important immunological strategies against *S. pneumoniae*.

### Evaluation of vaccine efficacy and the vaccination strategy for the elderly

Although pneumococcal pneumonia-related mortality or a pneumococcal pneumonia event would be desirable primary endpoint in clinical trials, a large sample size is required such as in the CAPiTA study to evaluate these endpoints.^52^ However, it is not feasible to gather a large sample size from the perspectives of costs and efforts. Therefore, surrogate markers are considered more realistic, although time point of antibody level measurement after vaccination also remains controversial.^88^ In clinical trials of pneumococcal vaccines, IgG and OI are usually used as surrogate markers to evaluate vaccine efficacy.^89^ Generally, OI, reflecting the functional ability of IgG to opsonize *S. pneumoniae* has been considered to be appropriate indicator for evaluating vaccine efficacy, rather than quantitative IgG measurement.

In our evaluation of serotype-specific IgG antibodies and serotype-specific OIs between PPV23 and PCV7 for pneumococcus vaccine-naïve elderly people (≥80 years of age),^90^ both PPV23 and PCV7 elicited increases in IgG and OI. Meanwhile, PCV7 was more potent than PPV23 in terms of its immunogenicity against 4 serotypes out of the 7 included in PCV7 (serotypes 4, 9V, 18C, and 23F). The safety of these preparations in these elderly individuals was also demonstrated, with no serious adverse effects observed in either group. A greater functional immune response with PCV13 than PPV23 as reported in the current study has also been reported regarding the majority of serotypes covered by PCV13 among adults aged >50 years.^53,54,91,92^

Based on these clinical findings, PCV13 could elicit more potent immunogenicity than PPV23 among the elderly. Furthermore, previous studies of successive immunization with PPV23 and PCV13 have already demonstrated that PCV13 vaccination followed by PPV23 is advantageous from an immunological point of view, as recommended by the ACIP.^55,57,93^ Concretely, initial immunization by PCV13 cause T-cell priming and subsequent PPV23 vaccination results in a booster effect even though PPV23 does not elicit T-cell activation. On the other hand, since initial immunization by PPV23 does not elicit T-cell priming, a booster effect of subsequent PCV13 vaccination is estimated to be weaker.

### Gap between immunological efficacy and vaccine strategy

As we have previously discussed regarding immunological efficacy, PCV13 vaccination alone or PCV13 vaccination followed by PPV23 elicits more potent immunogenicity than PPV23. This T cell mediated immune response is more dispensable for elderly people, who are under immunosenescence, in order to evoke sufficient immunogenicity.

In addition to this immunological theory, vaccine strategies need to consider the regional health care system, cost effectiveness, and serotype epidemiology. Vaccination against childhood diseases are considered to be cost effective, but some vaccinations for the elderly are still under debate. ^94-97^ For instance, the Joint Committee of the Japanese Respiratory Society and Japanese Association for Infectious Diseases do not recommend the routine use of PCV13 for adults due to the lack of currently available evidence regarding cost effectiveness in Japan, despite the existence of the evidence in other countries.^98-100^ In Japan, the gap between immunological efficacy and domestic vaccine strategies should be remembered, and clinical study in high quality to fill this gap is strongly warranted.

### Prerequisite for decision-making of pneumococcal vaccination policy –cost effectiveness

A discussion of the cost effectiveness of the 2 pneumococcal vaccines is essential, especially for making decisions regarding their introduction. This notion is supported by the United States Institute of Medicine, which stated that health economic deliberation plays an essential role in the prioritization of future vaccine strategies.^101^ Regarding pneumococcal vaccines, there have been some previous studies favoring the cost-effectiveness of PCV13; however, cost-effectiveness studies directly comparing PPV23 and PCV13 in the elderly are still limited.^98,102^ In general, the outcome of cost-effectiveness studies depends on several factors, such as the estimation of the vaccine effectiveness, proportion of people who are vaccinated, serotype epidemiology, estimation of the change in serotype distribution, baseline probability of infection, and medical expenses in the specific region.^99^ From this standpoint, the present results could not be fully applied to every region. Furthermore, these cost-effectiveness studies were sensitive to the assumption that PCV13, but not PPV23, is effective against non-bacteraemic pneumococcal pneumonia, which is different from the results of the CAPAMIS study. As supported in the CAPAMIS study or the clinical trials conducted in Japan, if PPV23 is somewhat effective against non-bacteraemic pneumococcal pneumonia, these outcomes showing superiority of PCV13 in terms of cost-effectiveness could differ considerably.^49,103,104^

When making decisions regarding vaccination policy, we should ideally calculate the cost-effectiveness for each region as well as referring to previous studies. However, some regions and countries do not have accurate epidemiological data, which is needed for studies regarding efficacy and cost-effectiveness of the vaccines. Even in Japan, data regarding the precise pneumococcal serotype distribution has been unavailable until quite recently. This shortage of epidemiological data has been an obstacle for vaccine introduction.^105^ In addition, tight budgets for immunization have hindered vaccine introduction, especially in low- and middle-income countries. To overcome these social problems, future collaborative program among international partners will play an important role as a new way of decision-making.^106^

## Conclusions

With the global aging society, to take appropriate measures regarding pneumococcal diseases in the elderly becomes more important. To implement effective vaccination strategies, a comprehensive understanding of immunosenescence and translation of additional basic findings and clinical evidence to clinical practice are warranted. To this end, a multidisciplinary research approach focusing on cost effectiveness and epidemiology is particularly essential.
